# In vitro and in silico evaluation of the serrapeptase effect on biofilm and amyloids of *Pseudomonas aeruginosa*

**DOI:** 10.1007/s00253-023-12772-1

**Published:** 2023-09-23

**Authors:** Georgios Katsipis, Dimitrios I. Avgoulas, George D. Geromichalos, Maria Petala, Anastasia A. Pantazaki

**Affiliations:** 1 Laboratory of Biochemistry, Department of Chemistry, Aristotle University of Thessaloniki, 54124 Thessaloniki, Greece; 2 Center for Interdisciplinary Research and Innovation, Laboratory of Neurodegenerative Diseases (LND), Thermi, 57001 Thessaloniki, Greece; 3 Laboratory of Chemical and Environmental Technology, Deparment of Chemistry, Aristotle University of Thessaloniki, 54 124, 54124 Thessaloniki, Greece; 4 Department of General and Inorganic Chemistry, Faculty of Chemistry, Aristotle University of Thessaloniki, 54124 Thessaloniki, Greece; 5 Laboratory of Environmental Engineering & Planning, Department of Civil Engineering, Aristotle University of Thessaloniki, 54124 Thessaloniki, Greece

**Keywords:** *Pseudomonas aeruginosa*, Biofilm, Serrapeptase, Functional amyloids, Viability, In silico molecular docking

## Abstract

**Abstract:**

*Pseudomonas aeruginosa* is an emerging threat for hospitalized and cystic fibrosis patients. Biofilm, a microbial community embedded in extracellular polymeric substance, fortifies bacteria against the immune system. In biofilms, the expression of functional amyloids is linked with highly aggregative, multi-resistant strains, and chronic infections. Serrapeptase (SPT), a protease possessing similar or superior anti-microbial properties with many antibiotics, presents anti-amyloid potential. However, studies on the employment of SPT against *Pseudomonas* biofilms and Fap amyloid, or the possible mechanisms of action are scarce. Here, SPT inhibited biofilm formation of *P. aeruginosa* ATCC 27853 on both plastic and glass surfaces, with an IC_50_ of 11.26 µg/mL and 0.27 µg/mL, respectively. The inhibitory effect of SPT on biofilm was also verified with optical microscopy of crystal violet-stained biofilms and with confocal microscopy. Additionally, SPT caused a dose-dependent decrease of bacterial viability (IC_50_ of 3.07 µg/mL) as demonstrated by MTT assay. Reduction of bacterial functional amyloids was also demonstrated, employing both fluorescence microscopy with thioflavin T and photometrical determination of Congo-red-positive compounds. Both viability and functional amyloids correlated significantly with biofilm inhibition. Finally, in silico molecular docking studies provided a mechanistic insight into the interaction of SPT with FapC or FapD, proving that both peptides are possible targets of SPT. These results offer new insights into the biofilm formation of *P. aeruginosa* and potentiate the involvement of SPT in the prevention and eradication of *Pseudomonas* biofilms.

**Graphical abstract:**

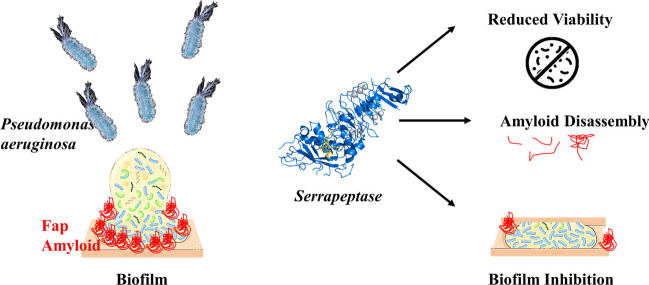

**Key points:**

• *Serrapeptase inhibits biofilm formation of P. aeruginosa on plastic and glass.*

• *Biofilm inhibition correlated with reduced viability and functional amyloid levels.*

• *In silico studies indicated that serrapeptase may target FapC and FapD peptides.*

**Supplementary Information:**

The online version contains supplementary material available at 10.1007/s00253-023-12772-1.

## Introduction

*Pseudomonas aeruginosa* is a Gram-negative, non-fastidious, facultative anaerobic bacterial species, with low nutritional requirements. Thus, it can colonize most surfaces, with a preference for moist installations like hospital room sinks, toilets, showers, patient care equipment (respiratory ventilators, contact lenses, dialysis tubes), and even soaps, hand creams, and disinfectants. Though mainly considered an abiotic inhabitant, *P. aeruginosa* was also found as part of the human flora of gut, arms, perineum, throat, and nose, and can also become an opportunistic pathogen (Moore and Flaws 2011; Pachori et al. 2019). Also, the lung of most of cystic fibrosis (CF) patients are typically infected with *P. aeruginosa*, accompanied by poor response to antimicrobial therapy (Lyczak et al. 2002).

Strains belonging to *P. aeruginosa* are at the core of healthcare-associated infections (HAI), leading to endemics of high morbidity. It is reported that 10% of all HAI are attributed to *P. aeruginosa*, and mainly affect immunosuppressed and intensive care unit patients, with severe cases of pneumonia and bacteremia (Raman et al. 2018; Pachori et al. 2019). Wistfully, 32.1% of *P. aeruginosa* isolates in Europe were reported resistant to at least one of the regular antimicrobial groups (ECDC 2019). Indeed, a group of the so-called “high-risk clones” of multidrug-resistant *P. aeruginosa* poses an extreme threat to be treated with urgency, as no available therapies are at hand. Also, previous antibiotic therapies are now blamed for the emergence of carbapenem-resistant *P. aeruginosa*. As a result, the implementation of non-antibiotic treatments and of mixed therapies to minimize the employed titers of antibiotics, are considered is imperative (Raman et al. 2018).

Contaminated tap water, sinks, plumbing systems, etc. are familiar sources for the resistant microbial spread of HAI from *P. aeruginosa*, usually due to cross-contamination from wastewater and poor hygiene protocols. In these spots, stagnant water is blamed for being a natural incubator for *P. aeruginosa* biofilm growth (Breathnach et al. 2012; Quick et al. 2014; Bicking Kinsey et al. 2017). Biofilm is a microbial community embedded in an extracellular polymeric substance (EPS) and is considered the dominant mode of microbial life (Stoodley et al. 2002). EPS is mainly composed of polysaccharides, proteins, lipids, and nucleic acids, providing the microbes with a well-hydrated environment for three-dimensional growth. Biofilms protect bacteria from ultraviolet radiation, extreme temperature, pH, salinity, and antibacterial factors. Besides the presence of EPS as a physical barrier, biofilm physiology can contribute to gaining resistance to multiple antibiotics (Yin et al. 2019). Among the several molecular components that are crucial for bacterial adaptability and biofilm formation, functional amyloids—a group of loosely ordered peptides—are recognized in both bacteria and fungi to be implicated in virulence and biofilm formation (Van Gerven et al. 2018). Amyloids are characterized by a signature motif of usually extracellular, elongated, unbranched fibers consisting of stacked β-sheets. These formations are highly resistant to protease treatment and are routinely identified by their ability to bind the dyes thioflavin T (ThT) and Congo red (CR) (Eisenberg and Jucker 2012; Taglialegna et al. 2016). Active metabolism is recognized as imperative for functional amyloid regulation and biofilm establishment (Reshamwala and Noronha 2011).

The functional amyloid of *P. aeruginosa* (Fap) has been recognized by its ability to bind both ThT and CR (Larsen et al. 2007; Dueholm et al. 2010). Fap fibers (amyloid-like fimbriae, ALF) are coded by the *fap* operon (*fap*ABCDEF), which is responsible for the expression of six different constituents of ALF. Fap subunits are chaperoned and processed at the extracellular space by the activity of FapA, D, and F, where polymerization is performed on the surface of the bacterial cell wall. FapC is recognized as the main component of these fibers, while FapB acts as an amyloid nucleator for FapC polymerization. ALF are considered essential for the biofilm formation of *P. aeruginosa*, and *fap* expression leads to bacterial aggregation and increased biofilm formation (Dueholm et al. 2013; Rouse et al. 2016; Rasmussen et al. 2023). ALF are highly resistant to high temperatures, dryness, and detergents and thus provide bacteria with the proper environment for survival in impossible environmental and in-host circumstances (Dueholm et al. 2010; Zeng et al. 2015).

Serrapeptase (SPT) is a metalloprotease discovered in the Gram-negative bacterium *Serratia marcescens* (Miyata et al. 1970). Multiple beneficial properties have been attributed to SPT, namely anti-inflammatory, analgesic (Tachibana et al. 1984; Nakamura et al. 2003; Al-Khateeb and Nusair 2008), anti-amyloid (Fadl et al. 2013; Metkar et al. 2017, 2020) and anti-biofilm properties (Longhi et al. 2008; Artini et al. 2011; Papa et al. 2013; Selan et al. 2015, 2017; Zapotoczna et al. 2015; Tsitsa et al. 2016). SPT is a promising agent for pharmaceutical applications, as it is non-toxic for animal cells (Chopra et al. 2009; Papa et al. 2013; Selan et al. 2017). Also, SPT may boost antibiotic efficacy in humans (Passariello et al. 2012; Sannino et al. 2013) and rats (Mecikoglu et al. 2006), presenting no side effects during its employment, except for some gastrointestinal disturbances. Though reduced bioavailability was highlighted as a key drawback for the medical employment of SPT (Jadhav et al. 2020), SPT-loaded nanoparticles, liposomes, gels, etc. can be employed for optimizing SPT's in vivo administration (KV et al. 2008; Shinde and Kanojiya 2014; Devlin et al. 2021). However, the possible anti-biofilm effect of SPT on *P. aeruginosa* and its mechanistic details has been scarcely studied (Artini et al. 2022).

We have previously proven that SPT can be exploited against biofilm formation from both susceptible and multi-drug resistant *Staphylococcus aureus* strains; furthermore, we have demonstrated the effect of SPT against the bacterial viability, amyloids, and virulence factors (Katsipis and Pantazaki 2023). Here, the anti-biofilm activity of SPT against *P. aeruginosa* ATCC 27853, a bloodborne model-strain with multiple antibiotic resistances (Cao et al. 2017), was verified in a semi-quantitative manner. In the present study, we also attempted to evaluate the biofilm formation from *P. aeruginosa* and the effect of SPT on it with diverse methods, e.g., plastic tissue culture plate (TCP) method and optical, fluorescence, and confocal microscopy. As far as we know, the anti-biofilm activity of SPT against this bacterial strain has not been reported before. Furthermore, the inhibitory effect of SPT on bacterial viability/metabolic activity is also demonstrated. The effect of SPT on functional amyloids in terms of ThT- and CR-positive compounds has been also studied, while in silico molecular docking calculations on the interaction of SPT with crucial peptides FapC and FapD provide probable further insights on the anti-amyloid potential of SPT. Statistical analyses have also been used to study the interrelation of *Pseudomonas* viability and amyloid titers with biofilm formation. The present results should offer novel possibilities for introducing SPT in clinical practice and demonstrate a mechanistic model for its activity against biofilm and amyloids of *P. aeruginosa*.

## Materials and methods

### Reagents and microbial strains

SPT was purchased as capsules of 60,000 IU (Health Aid LTD, UK). The powdered interior of the capsules was mixed with growth medium (1 capsule interior per 10 mL medium), vortexed, and filtered (Minisart NY 25, sterile 0.45-µM filters, Sartorius Stedim Biotech GmbH, Goettingen, Germany) to abort non-dissolved materials and excipients. No further purification process was employed. The SPT concentration of the received solutions was estimated with the Bradford-Bearden assay, as modified by Zor and Selinger (Bearden 1978; Zor and Selinger 1996). At the same time, the proteolytic efficiency of the preparations was tested with the protease substrate azocasein (A-2765, Sigma-Aldrich, St. Louis, IL, USA), as previously described (Vélez-Gómez et al. 2019). Tryptone (#403682), dimethyl-sulfoxide (DMSO) (#D5879), and (3-[4,5-dimethylthiazol-2-yl]-2,5 diphenyl tetrazolium bromide) (MTT) (#A2231) were purchased from PanReac AppliChem (Darmstadt, Germany). Soybean peptone (#70,178), crystal violet (CV) (#C0775), ThT (#T3516), 4′,6-Diamidino-2-phenylindole dihydrochloride (DAPI) (#D9542), and CR (#75768) were purchased from Sigma-Aldrich (St. Louis, MO, USA). Sterile double-distilled H_2_O was used throughout the experimental procedures. *P. aeruginosa* strain ATCC® 27853™ (Boston 41501) was used in this study.

### Growth conditions and biofilm formation

All growth media were autoclaved before use. Bacteria were stocked at 20°C in Luria–Bertani (LB) medium (% w/v: 1 tryptone, 0.5 NaCl, 0.5 yeast extract) containing 20% (v/v) filtered glycerol. Initial cultures were received with the suspension of 100 µL of stock culture in 10 mL of LB medium and grown overnight, at 37°C, in a shaking incubator. The composition of the employed medium for biofilm growth was defined after pilot tests with several dilutions of LB or Tryptic Soy Broth (TSB) medium for the determination of planktonic growth, biofilm formation, and functional amyloids (see the following paragraphs of the “Materials and methods” section for the determination protocols). Biofilm formation was finally performed with a 4 times diluted TSB (% w/v: 0.425 tryptone, 0.075 soybean peptone, 0.125 NaCl, 0.0625 Κ_2_HPO_4_), supplemented with 0.25% (w/v) glucose, for 24 h, at 37 °C, at static conditions.

For the semi-quantification of biofilm formation, bacteria were grown in polystyrene (PS) plastic surface of 96-well TCP, in the presence (Treated) or absence (Control) of SPT, while for microscopy evaluation bacteria were grown on glass microscopy slides (1 × 1 cm). Overnight LB cultures were diluted 20 times with biofilm medium and transferred in 96-well TCPs (#CLS3997, Corning, New York. USA) for TCP method, or petri dishes for microscopy evaluation, respectively. Growth on glass slides was performed by immersing the slides in the diluted bacteria in the petri dishes. For planktonic bacteria estimation, the cultures’ turbidity was read at 630 nm in a microplate reader (BioTek Instruments Inc., USA). Biofilm determination on the surface of the TCP wells was performed after dyeing the cells with CV, as previously described (Katsipis et al. 2021). Microscopy evaluation of CV-stained glass slides was done after fixing the biofilms with 2.5% (v/v) glutaraldehyde for 15 min, followed by staining with 0.4% (w/v) CV for 10 min. Stained biofilms were then photographed under the bright field of a Nikon Eclipse Ci fluorescence microscope.

### Biofilm evaluation by confocal microscopy

Analysis of biofilm-coated surfaces was performed according to standard ISO 25178 using a 3D Optical Surface Metrology System Leica DCM8 device. The biofilm-coated surfaces, once removed from the incubation test cells, were washed off with PBS solution. Afterward, glutaraldehyde solution (2.5%, v/v) was applied to the surface biofilm fixation as mentioned above, enabling the analysis of its morphology. For each biofilm-coated surface, three images were obtained (× 10 magnification), corresponding to a surface of 1.3 mm × 1.6 mm for each image. Images were subjected to analysis using the Leica Map software (LeicaGmbH, Germany), and the parameters of the roughness profile (Sdr, %), the maximum height of peaks (Sp, µm), and the arithmetic mean height of surface (Sq, µm) were determined using the acquired data.

### Determination of bacterial viability by MTT assay

MTT assay is now accepted as an effective method for estimating bacteria viability and metabolic activity (Wang et al. 2010; Grela et al. 2018). *P. aeruginosa* bacteria were grown in the presence or absence of SPT, in glass tubes sealed with a gauze cap filled with hydrophobic cotton, and with the biofilm conditions described before. After growth, bacteria were harvested by centrifugation (5,000 rpm for 10 min) and then washed and resuspended in PBS. The viability of the bacteria cells was determined with 0.5 mg/mL ΜΤΤ (final concentration), after incubation at 37°C, for 30 min, under mild shaking. A sample containing PBS and the MTT substrate was also prepared as blank. The formazan crystals received due to MTT reduction were then centrifuged and dissolved with DMSO, and the absorbance was read at 570 nm in a microplate reader. The bacterial viability/metabolic activity was finally normalized against the total bacterial turbidity of the PBS-resuspended cell samples and was expressed by setting the viability of the untreated bacteria at 100%, as previously described (Wang et al. 2010).

### Estimation of functional amyloid content with Thioflavin T

In order to study the distribution of functional amyloids on the biofilm surface, as well as the effect of SPT treatment on them, *P. aeruginosa* was grown on glass slides and then fixed with glutaraldehyde. Afterward, slides were covered with a PBS solution containing 10 µΜ ThT for amyloid staining and 1 µg/mL DAPI for general EPS staining due to extracellular DNA complexation. Staining was performed for 15 min in the dark, and then biofilms were washed thoroughly with PBS and observed under a Nikon Eclipse Ci fluorescence microscope equipped with a Color Camera Nikon DS-Fi3 (× 20, FITC filter, 80 ms exposure for ThT/ × 20, DAPI filter, 30 ms exposure for DAPI).

### Image processing

Image analysis was carried out employing the open-access software package Fiji (Image J, US National Institutes of Health, Bethesda, Maryland, USA). Briefly, regarding the processing, the experimental images were converted to greyscale, and subsequently, a threshold was set to create binary images separating the background from features of interest. Finally, analysis of particles was performed providing information about the total surface coverage (%) and their number. More specifically, concerning the fluorescence imaging processing, brightness, and contrast for green and blue channels, respectively, were also adjusted using Fiji, while both channels were merged to generate composites. For each biofilm-coated surface, at least 6 images corresponding to different points of the surface were obtained and further analyzed statistically.

## Estimation of functional amyloid content with Congo red

*P. aeruginosa* amyloids were estimated with the CR assay, as previously described (Reichhardt et al. 2015). Briefly, bacteria grown in glass tubes under biofilm conditions as described above were received and mixed with a chill CR solution of 10 µg/mL (final concentration), in PBS. A sample in the absence of bacteria was also prepared to estimate the absorbance of the pure dye solution. Bacteria were incubated at room temperature for 10 min and then pelleted by centrifugation (13,500 rpm for 5 min). The absorbance of the supernatant for CR was then read at 500 nm with a Selecta 2005 UV–Vis spectrophotometer (cell path of 1 cm). The retained CR dye from bacteria corresponding to the amyloid compounds was inversely calculated, by subtracting the absorbance values from the ones received from samples containing only the dye solution. Finally, results were normalized versus the rough bacterial turbidity of the PBS-resuspended cell samples and were expressed by setting the amyloid content of the untreated bacteria at 100%.

### Molecular docking calculations

To elucidate the possible interaction between SPT and the FapD or FapC proteins, the pyDockWEB server (https://life.bsc.es/pid/pydockweb) was employed (Jiménez-García et al. 2013) for structural prediction of protein–protein interactions. Given the 3D coordinates of two interacting proteins, pyDockWEB returns the best rigid-body docking orientations generated by FTDock and evaluated by the pyDock scoring function, which includes electrostatics, desolvation energy, and limited van der Waals contribution. PyDockWEB server makes use of the SCWRL software for rebuilding missing side chains. PyDockRST software package uses the percentage of satisfied distance restraints, together with the electrostatics and desolvation binding energy, to identify correct docking orientations. This methodology drastically improves the docking results when compared to the use of energy criteria alone and is able to find the correct orientation within the top 20 docking solutions in 80% of the cases (Chelliah et al. 2006). From the output sorted by the pyDock scoring function, the top one hundred conformations was derived among a total of 500 conformations. From a table with a list of generated conformations scored and ranked by the energy scoring, the best protein complexes between SPT and FapC or, FapD were selected.

For FapC *Pseudomonas sp.* UK4 FapC amyloid-like fimbriae precursor protein was used, with accession coding EEP64551.1 of European Nucleotide Archive (ENA) Advanced Search API (https://www.ebi.ac.uk/ena/browser/home) in FASTA format for modeling and prediction of the protein by running the BLAST sequence similarity search by UniProt (https://www.uniprot.org/uniprotkb/C4IN70/entry) with very good Model Confidence (most of the structure displaying pLDDT values among 70 and 90 (Confident), while lower portions of the structure display either pLDDT > 90 (Very high) or 70 > pLDDT > 50 (Low) and pLDDT < 50 (Very low)) (Protein name: FapC amyloid-like fimbriae protein; Gene name: fapC; ORF name: PSUK4_00030; Organism name: Pseudomonas sp. UK4; Taxonomic identifier: 452,680 NCBI; Length 250; Mass (Da) 24,998). The AlphaFold structure prediction database (either from the AlphaFoldDB databases or the UniProt databases) was downloaded for the FapC 3D structure model AF-C4IN70-F1-model_v4.pdb (https://alphafold.ebi.ac.uk/entry/C4IN70).

On the other hand for FapD, the modeled after the homologous C39 peptidase domain of ABC transporter PCAT1 was employed, PDB: 4RY2 (FapD model of conserved core domain) (Dueholm et al. 2010).

### Statistical analyses

All statistical analyses and graph constructions were done with GraphPad Prism 8 (GraphPad Software Inc.). Provided graphs include bars representing mean values ± standard error of the mean (SEM), from at least three independent experiments. The possible statistical significance for differences between the untreated (control) and treated samples using increasing SPT concentrations, was examined with standard one-way analysis of variance (ANOVA), after correcting for multiple comparisons with Dunnett’s test. Before performing ANOVA, the normality of the replicates was verified with Anderson–Darling, D'Agostino-Pearson omnibus, Shapiro–Wilk, and Kolmogorov–Smirnov tests, while possible significant differences between the standard deviations of the various samples were evaluated with Brown-Forsythe test. Inhibitions at 50% of the untreated sample (IC_50_) and the corresponding 95% of confidence intervals – CI (95%) were calculated based on the equation: *log [SPT] vs. normalized inhibition (%), with variable slope.* Possible correlations were examined with Pearson’s analysis, determining r coefficients and two-tailed *p* values. Statistically significant results were considered for *p* < 0.05. Notations for statistically significant differences between control (untreated) and treated samples: *p < 0.05; **p < 0.01;*** *p* < 0.001; and ****p < 0.0001.

## Results

### The effect of growth medium composition on biofilm formation of *P. aeruginosa*

The composition of the growth medium was standardized in terms of efficient biofilm growth. In the current growth conditions and the LB medium, *P. aeruginosa* has mainly adapted to the planktonic growth mode, as proven by the very low CV staining. To that notice, the LB medium was not studied further. TSB medium supplemented with 1% (w/v) glucose was evaluated for biofilm formation. Additionally, two different TSB dilutions were also analyzed: 2 times and 4 times diluted. The planktonic bacteria growth and the levels of amyloids were also analyzed for each medium dilution employed. The results for TSB are graphically depicted in Supplementary Fig. S1.

Increased biofilm formation was found at the highest studied dilution of the growth medium (4 times diluted TSB), implying that in the studied conditions, lower availability of nutrients induces a biofilm-growth phenotype for *P. aeruginosa* in a greater degree compared to a nutrient-rich environment. Increased levels of biofilm formation were accompanied by lower levels of planktonic bacteria growth, as demonstrated by the turbidity of the growth medium, and elevated titers of functional amyloids, as determined by CR staining. These results indicate the induction of functional amyloid expression and biofilm formation under lower availability of nutrients. Consequently, 4 times diluted TSB was employed for all further experimentations.

### SPT inhibits the biofilm formation of *P. aeruginosa* on plastic

**Fig. 1:**
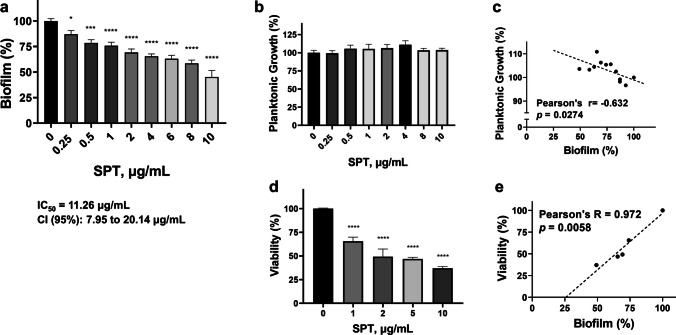
Inhibition of biofilm formation of *Pseudomonas aeruginosa* by serrapeptase (SPT) and effect on planktonic bacteria. *P. aeruginosa* ATCC 27853 bacteria were grown under static conditions in tissue culture plates (TCPs) in the presence or absence of SPT. Biofilm formation **(a)** was semi-quantified with crystal violet staining after dye extraction and read at 570 nm, while the levels of planktonic bacteria **(b)** were estimated by determination of medium turbidity at 630 nm. Biofilm inhibition correlates inversely with the rough density of planktonic bacteria **(c)**. *P. aeruginosa* ATCC 27853 bacteria grown under static conditions in glass tubes, in the presence or absence of SPT, were collected and analyzed for their ability to reduce (3-[4,5-dimethylthiazol-2-yl]-2,5 diphenyl tetrazolium bromide) (MTT). The levels are presented as % of the control (untreated) value **(d)**. Biofilm inhibition correlates with reduction in planktonic bacteria viability **(e)**. Bars represent mean values ± SEM from at least three independent experiments, with the value of the untreated bacteria culture (control) set at 100%. Standard ANOVA with Dunnett’s correction for multiple comparisons was employed for the statistical analysis. Notations for statistically significant differences between control (untreated) and treated samples: *p < 0.05; **p < 0.01; ***p < 0.001; ****p < 0.0001

The ability of various SPT concentrations to inhibit the formation of *P. aeruginosa* biofilm on TCP wells was studied under static conditions. Bacterial biofilms were stained with CV—a dye that binds mainly on polysaccharides like peptidoglycans (Jones 2017) and semi-quantified after the extraction of the dye and its determination at 570 nm. After normalization of the data, the ability for biofilm formation (% of the control sample) on plastic surfaces of TCP is presented in Fig. 1a for indicative SPT concentrations of 0.25 to 10 µg/mL. The analysis with the TCP method demonstrated that SPT is a potent inhibitor for biofilm formation of *P. aeruginosa* on plastic surfaces, presenting a maximum inhibition at 10 µg/mL of SPT (-55%) and a calculated IC_50_ value of 11.26 µg/mL (CI 95%: 7.95 to 20.14 µg/mL).

To further demonstrate the anti-biofilm efficacy of SPT, the bulk of planktonic bacteria were estimated at the growth medium by measuring its turbidity at 630 nm. The levels (% of the control sample) of planktonic bacteria for each SPT treatment and the control are presented in Fig. 1b. Increasing doses of SPT lead to higher floating bacteria titers. However, statistical significance could not be verified. However and in agreement with biofilm diminishment under SPT treatment, a negative, significant correlation between biofilm formation and planktonic bacteria turbidity was verified (Fig. 1c), indicating that SPT treatment possibly prevented the ability for growth under biofilm conditions and led to higher numbers of planktonic bacteria. However, it is not possible to determine by this turbidity measurement if the floating bacteria were viable and thus, a viability assay was also employed.

### SPT treatment impairs the viability/metabolic activity of *P. aeruginosa*

MTT is reduced mainly by oxidoreductive enzymes of the oxidative metabolism of viable bacteria (Grela et al. 2018) and thus can be exploited for studying the effect of SPT on *P. aeruginosa* viability and metabolic capability. Bacteria were grown under biofilm conditions, collected, and then examined for their ability to actively reduce MTT. After normalization, test results are summarized in Fig. 1d.

SPT treatment causes a significant decline in the metabolic efficiency of *P. aeruginosa* bacteria in a dose-dependent manner, with a calculated IC_50_ value of 3.07 µg/mL (CI 95%: 2.26 to 4.13 µg/mL). The maximum viability inhibition was -63% when 10 µg/mL of SPT was employed. Correlation analysis also indicated that impairment of metabolic viability coincides with biofilm inhibition, as a significant positive correlation was found (Fig. 1e). These results demonstrate that the presence of SPT can hijack proper bacterial metabolism, leading to impaired viability. This effect can and possibly sensitizes bacteria and thus presents a promising potential for antimicrobial therapy.

### SPT inhibits the biofilm formation of *P. aeruginosa* on glass

**Fig. 2:**
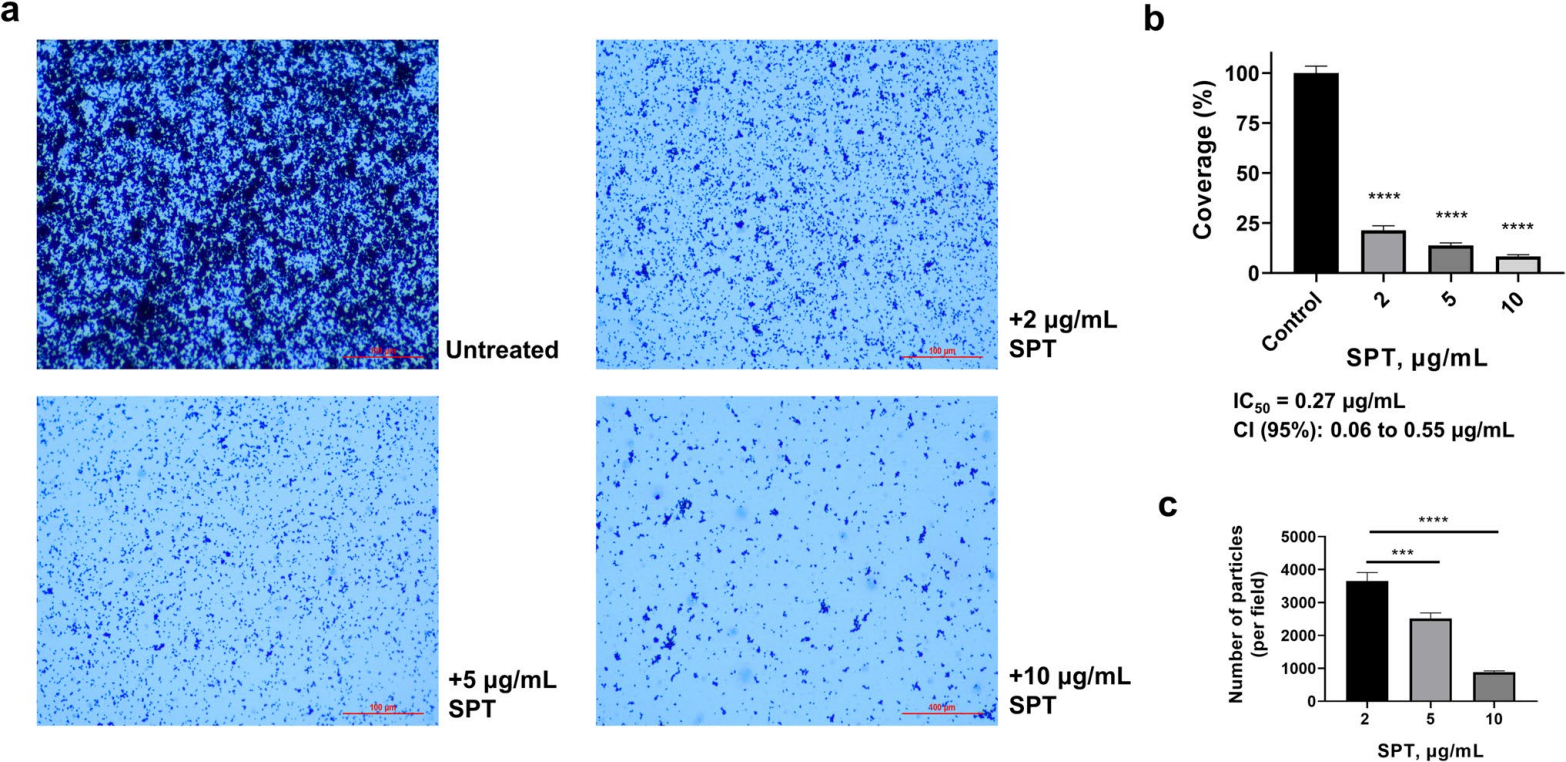
*Pseudomonas aeruginosa* biofilms formed on glass slides in the absence (untreated) or presence of SPT concentrations (2, 5, and 10 µg/mL), after fixation and staining with crystal violet. Bright images **(a)** are received from Nikon Eclipse Ci fluorescence microscope. Densitometric analysis of the coverage of glass surface **(b)** and number of particles for different SPT concentrations **(c)** were estimated from analysis of the received images with Image J. Due to the dense biofilm network of untreated biofilms, particle analysis from untreated samples were omitted from the given graph and statistical analyses were performed versus the lower employed dose of 2 µg/mL SPT. Bars represent mean values ± SEM from at least three independent experiments. Standard ANOVA with Dunnett’s correction for multiple comparisons was employed for the statistical analysis. Notations for statistically significant differences: *p < 0.05; **p < 0.01; ***p < 0.001; ****p < 0.0001

The ability of SPT to inhibit the formation of *P aeruginosa* biofilm on glass was also studied. Bacterial biofilms grown on microscopy slides were stained with CV and then observed and photographed under a bright field. Representative images received from the analysis are provided in Fig. 2a. Untreated *P. aeruginosa* cultures form a dense network consisting of bacterial aggregates and intermediate abiotic areas, that probably represent water channels for biofilm active nourishment. However, treatment with 2, 5, or 10 µg/mL of SPT dramatically disrupted biofilm structure, with only minimal bacterial assemblies observed at the highest employed dose. Densitometric analysis on the received images from CV-stained biofilms further supports the anti-biofilm ability of SPT, proving that protease treatment can abruptly impair biofilm colonization on the glass surface (IC_50_ = 0.27 µg/mL, CI 95%: 0.06 to 0.55 µg/mL) (Fig. 2b). A maximal inhibition of -92% was recorded at 10 µg/mL of SPT treatment. Additionally, SPT led to a significant dose-dependent decrease of the microbial aggregates, in terms of calculated particles on the observed fields (Fig. 2c). It should be noted that due to the dense biofilm network of untreated biofilms, it was technically problematic to estimate the rough number of microbial aggregates with the particle analysis, so the results from untreated samples were omitted from the given graph and statistical analyses were performed versus the lower employed dose of 2 µg/mL SPT. These results prove the preventive nature of SPT against biofilm formation on glass surfaces, which, based on the current results of the TCP method, surpasses the corresponding effect for plastic surfaces.

**Fig. 3:**
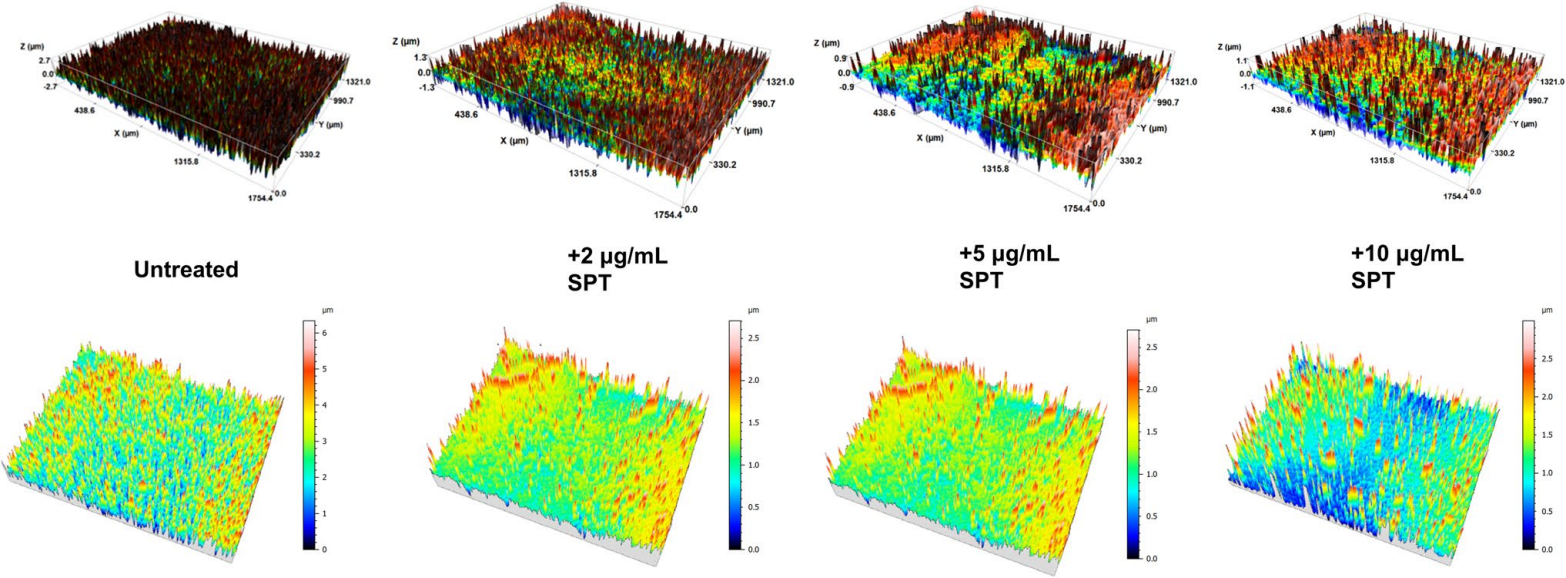
3D images of *Pseudomonas aeruginosa* biofilms on glass microscopy slides, grown in the absence (untreated) or presence of SPT concentrations (2, 5, and 10 µg/mL), obtained by confocal microscopy (3D Optical Surface Metrology System Leica DCM8)

Confocal microscopy of fixed *Pseudomonas* biofilms on glass slides provided further understanding of the effect of SPT and representative analyses graphs are provided in Fig. 3. The addition of SPT induced significant modifications in the morphology of biofilms on glass. 3D images suggested a much thicker and denser biofilm matrix with numerous peaks higher than 3 µm and various scattered peaks greater than 5 µm. SPT treatment provoked the disruption of biofilm formation and structure formulation. As shown in Fig. 3, biofilm thickness was considerably lowered after the application of 2 µg/mL SPT, while higher doses up to 10 µg/mL caused the formation of irregular structures with sporadic peaks that did not exceed 2.5 µm. Profilometric measurements over the majority of glass surfaces confirmed the reduction of surface roughness, in terms of developed surface area. Indeed, SPT increases up to 5 µg/mL minimize surface developed area close to clean surface. Likewise, maximum height of peaks and mean height of the surface were drastically reduced even after treatment with just 2 µg/mL SPT (Supplementary Figures S2 and S3).

### SPT treatment reduces functional amyloid levels of *P. aeruginosa*

**Fig. 4:**
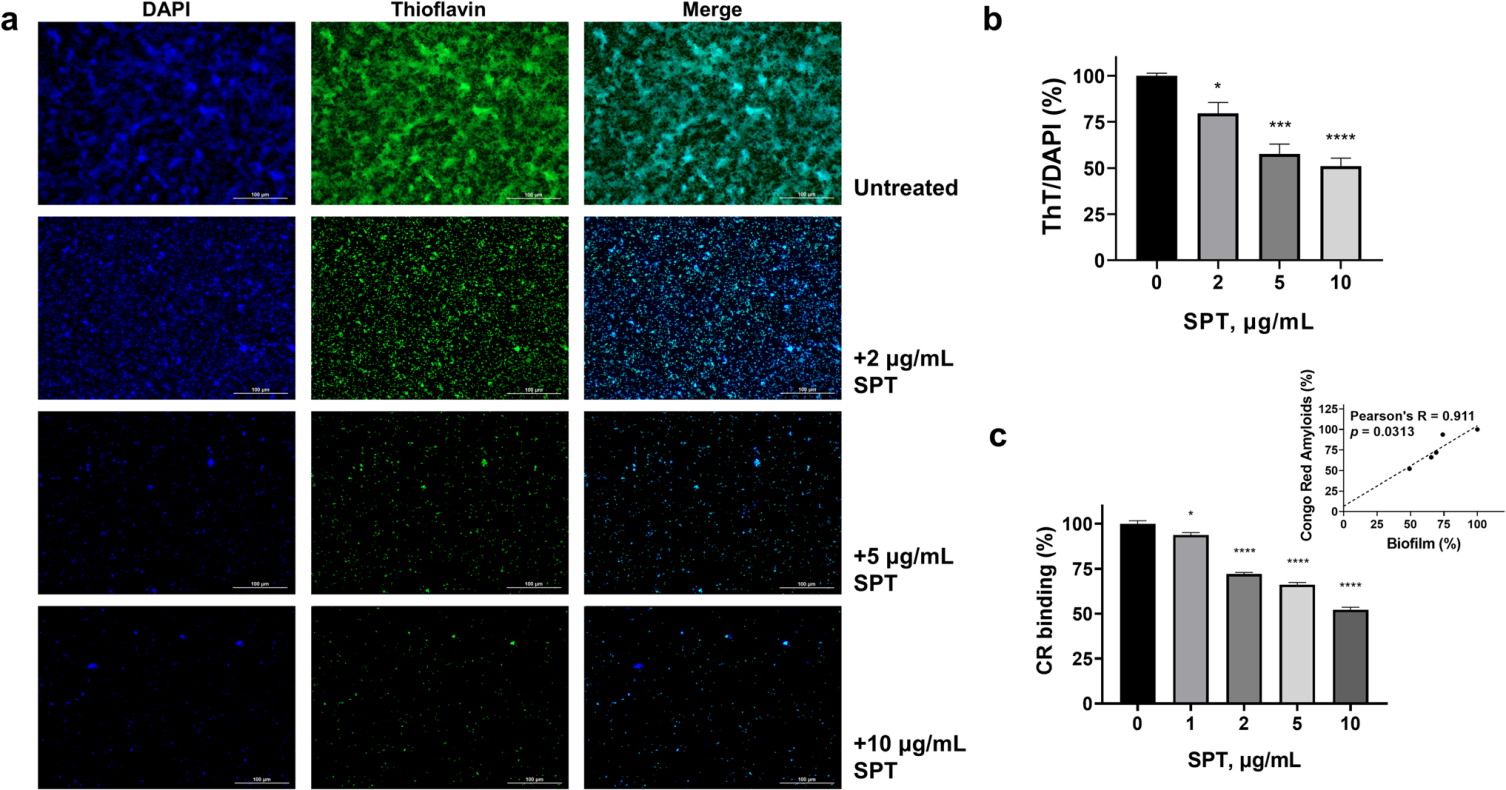
Effect of serrapeptase (SPT) on levels of functional amyloids of *P. aeruginosa* bacteria. **(a)** *P. aeruginosa* ATCC 27853 bacteria were grown under static conditions on glass coverslips, in the absence (untreated) or presence of different SPT concentrations, were fixed with glutaraldehyde, and stained with thioflavin T (ThT) for functional amyloids and 4′,6-diamidino-2-phenylindole (DAPI) for rough staining of biofilm matrix due to extracellular DNA. Bacteria were then observed under a Nikon Eclipse Ci fluorescence microscope (× 20, FITC filter, 80 ms exposure for ThT, × 20, DAPI filter, 30 ms exposure for DAPI) and the density of the stained cells was quantified with Image J **(b)**. *P. aeruginosa* ATCC 27853 bacteria were grown under static conditions in tubes in the presence or absence of SPT and were collected and mixed with CR solution. The retained dye, which corresponds to the levels of expressed functional amyloids, was inversely estimated by reading the absorbance of the supernatant at 500 nm, after discarding bacteria with centrifugation. The levels are presented as % of the control (untreated) value **(c)**. Bars represent mean values ± SEM from at least three independent experiments, with the value of the untreated bacteria culture (control) set at 100%. Standard ANOVA with Dunnett’s correction for multiple comparisons was employed for the statistical analysis. Notations for statistically significant differences between control (untreated) and treated samples: *p < 0.05; **p < 0.01; ***p < 0.001; ****p < 0.0001. Pearson’s correlation analysis (inset of c) indicated a significant, positive correlation between biofilm formation and bacterial viability

The dyes CR and ThT were previously reported for employement in the determination of bacterial functional amyloids (Zeng et al. 2015; Reichhardt et al. 2015). Given the significance of Fap for aggregation and biofilm formation of *Pseudomonas* (Dueholm et al. 2013) and the anti-amyloid properties attributed to SPT (Metkar et al. 2017), the effect of SPT on *P. aeruginosa* amyloids grown under biofilm conditions was studied here. Cells were stained with the fluorescent dye ThT for amyloid detection and with DAPI as a general marker for biofilm EPS and observed under fluorescence microscope (Fig. 4a) or stained with CR and the retained dye was inversely estimated. The normalized results for the titers of ThT- and CR-positive compounds are presented in Fig. 4b and c, respectively.

Treatment with SPT leads to a dose-dependent reduction of functional amyloids of *P. aeruginosa* bacteria, with an IC_50_ value estimated at 10.07 µg/mL (CI 95%: 7.77 to 14.68 µg/mL) for CR-positive compounds, while ThT-positive compounds were also found to be reduced (in terms of ThT/DAPI ratio), with a similar IC_50_ of 8.64 µg/mL (CI 95%: 6.65 to 11.33 µg/mL). Maximum inhibition of amyloid levels was recorded at 10 µg/mL of SPT, at -48% to that of control for CR-positive compounds and at -52% for ThT-positive compounds. Correlation analysis (inset of Fig. 4c) indicated that amyloid reduction, as determined by CR retention, interrelates positively and significantly with biofilm inhibition. These results verify the importance of amyloids for adequate biofilm formation of *P. aeruginosa*, as a lower amyloid content in *P. aeruginosa* is directly linked with a lower capability for biofilm formation. Moreover, they underline the anti-amyloid activity of SPT and propose a possible mechanism for that. 

### Protein–protein molecular docking calculations

Since SPT acts against bacterial biofilms with a possible effect on amyloids of *P. aeruginosa* and, both FapC and FapD are supposed to be possible targets of SPT, it is most rational to seek any existing interaction between SPT and any of these two peptides to suggest a probable proteolytic mechanism. The total binding energies of the best binding pose predicted by pyDockWEB (conformation of first rank according to the computed binding energy) between SPT and FapC and FapD proteins (Table 1), were calculated taking into account the following energetic components: electrostatic, desolvation, and Van der Waals (VDW) (term weighted to 0.1 by default). The total binding energy represents the sum of the 3 previous energies. The proposed structural prediction models of two Fap fibril biogenesis, the major Fap amyloid component FapC amyloid–like fimbriae protein produced by the BLAST sequence similarity search by UniProt model of FapC (AF-C4IN70-F1-model_v4.pdb) and the FapD modeled after the homologous C39 peptidase domain of ABC transporter PCAT1 (PDB ID: 4RY2) are depicted in Supplementary Figure S4. For FapC the generated model demonstrated very good Model Confidence (most of the structure displaying pLDDT values between 70 and 90 (Confident), while lower portions of the structure display either pLDDT > 90 (Very high) or 70 > pLDDT > 50 (Low) and pLDDT < 50 (Very low)).

**Table 1:** Predicted binding energies (in kcal/mol) of SPT/FapD C39 and SPT/FapC (Binding Sites I and II) protein complexes

	SPT/FapC Binding Site I	SPT/FapC Binding Site II	SPT/FapD C39
Electrostatic	− 31.240	− 8.036	− 40.289
Desolvation	− 5.433	− 36.538	1.337
Van der Waals	1.885	8.361	10.134
Total	− 36.485	− 36.212	− 37.939

**Fig. 5:**
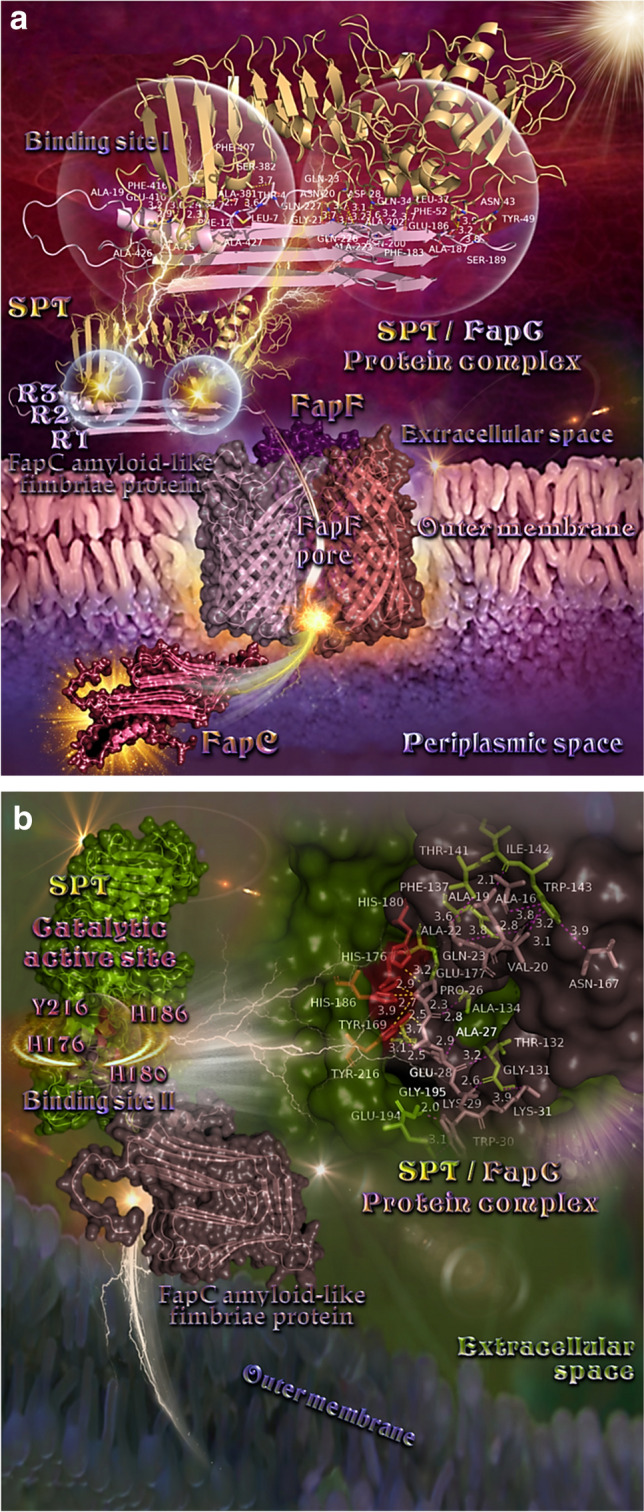
**(a)** A mechanistic model of the interaction between SPT and FapC demonstrated a possible binding/proteolytic activity of SPT on FapC. Binding architecture of FapC/SPT protein complex in the extracellular space at Binding Site I, after transportation of FapC (the main fibril component) out of the cell through the outer membrane using FapF trimeric β–barrel polypeptide membrane transporter via FapF pore. Proteins are depicted as cartoon colored by raspberry and light pink in the periplasmic and extracellular space, respectively (FapC), yellow–orange (SPT), and light pink, deep purple, and deep salmon (FapF for chains A, B, and C, respectively). FapC in the periplasmic space and FapF are additionally depicted with semi–transparent surface colored also by the cartoon colors. A close–up view of the Binding Site I mapping interactions of the best docking pose orientation, between SPT and FapC is also shown at the upper part of the figure, with the proteins depicted in cartoon representation and binding residues of both proteins illustrated in stick model colored according to cartoon. Binding contacts between SPT/FapC are indicated in yellow dotted lines. The final structure was ray–traced and illustrated with the aid of PyMol Molecular Graphics System. (**b)** A mechanistic model of the interaction between SPT and FapC at Binding Site II, demonstrating a possible binding/proteolytic activity of SPT on FapC. Binding architecture of FapC/SPT protein complex in the extracellular space at Binding Site II: the catalytic active site cleft ligated by Hisl76, Hisl80, Hisl86, and Tyr216. Proteins are depicted as cartoon colored by split pea green (SPT) and dirty violet (FapC) with additional depiction of semi–transparent surface colored also by the cartoon colors. The residues in the catalytic active site on the cartoon representation and the surface, are highlighted in firebrick color. A close–up view of the Binding Site II mapping interactions of docking pose orientation (at the upper right part of the figure), between SPT and FapC is also shown with the proteins depicted in semi–transparent surface representation and the binding residues of both proteins illustrated in stick model colored according to surface. Binding contacts between SPT/FapC are indicated in yellow dotted lines for SPT and purple for FapC. The final structure was ray–traced and illustrated with the aid of PyMol Molecular Graphics System

**Fig. 6:**
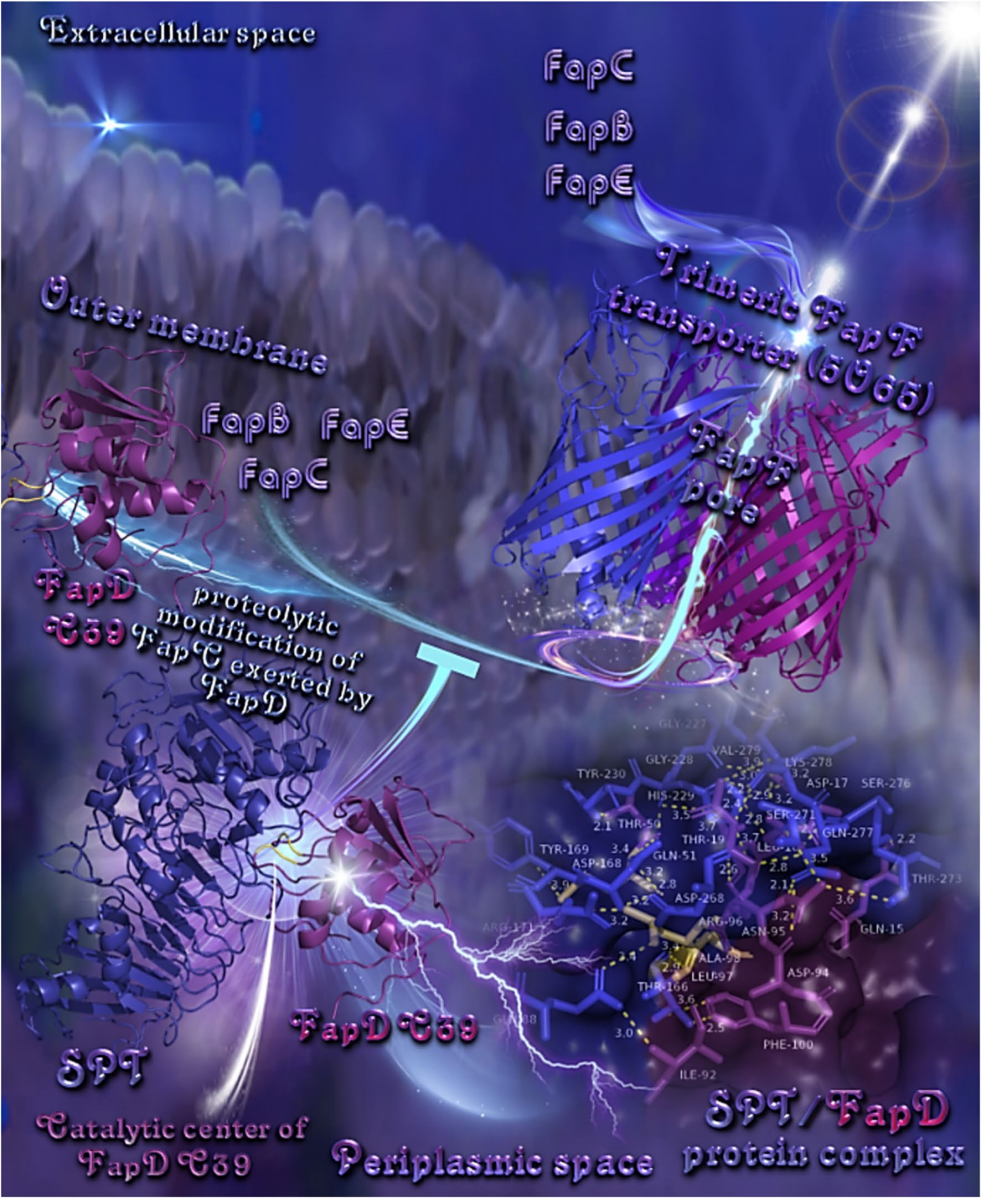
A mechanistic model of the biogenesis machinery of Fap in *P. aeruginosa* depicting the role of FapD in affecting the release of FapC to the extracellular space and the role of SPT binding to FapD on the FapC release. FapC passes through the outer membrane using FapF trimeric β–barrel polypeptide transporter (PDB: 5O67) via FapF pore. FapD, modeled after the homologous C39 peptidase domain of ABC transporter PCAT1 (PDB ID: 4RY2), is a protease involved in the proteolysis of Fap proteins and is shown to perform an essential proteolytic modification of FapC while residing in the periplasmic space between the inner and outer membrane, before its secretion from the cell. This activity may be affected by the SPT binding on FapD’s catalytic center, probably affecting its active peptidase function (the inhibitory activity of SPT on FapC release is shown by the inhibition arrow in the figure). The catalytic center of FapD is illustrated in the interface region between FapD and SPT in yellow–orange color. All proteins are depicted in cartoon colored in deep blue and violet purple for SPT and FapD, respectively, and in tv blue, purple blue, and deep purple for FapF (for chains A, B, and C, respectively). A close–up view of the binding site mapping interactions of the best docking pose orientation between SPT and FapD is also shown at the bottom right side of the figure, with binding residues of both proteins illustrated in stick model colored according to cartoon. Binding contacts between SPT/FapD are indicated in yellow dotted lines. The final structure was ray–traced and illustrated with the aid of PyMol Molecular Graphics System

The binding architecture models of protein–protein binding poses between SPT and FapC and between SPT and FapD are illustrated in Figs. 5 and 6. The docking predicts the formation of a variety of interactions between the components of the protein complexes including hydrogen bonds, hydrophobic, polar, π-polar, π-π displaced and T-shaped, π-alkyl hydrophobic, and π-anion charged electrostatic interactions (Supplementary Table S1 and Supplementary Table S2).

The Predicted Aligned Error (PAE) plot for FapC amyloid-like fimbriae protein indicating the AlphaFold’s expected distance position error at residue x, when the predicted and true structures are aligned on residue y, is depicted in Supplementary Figure S5. From Supplementary Table S1 it is deduced that most binding contact residues of FapC correspond to regions involving residues 12–19 and 183–226. From Figure S5, the alignment of these residues x (12–19, 183–226 when the prediction and true structure are aligned on residue y) results in confidence in the relative position of them. From the PAE plot, it is deduced that the predicted errors for FapC are restricted to very low levels (dark green shades indicating low errors).

The docking procedure revealed two binding sites of the docked proteins SPT and FapC, namely Binding Site I (Fig. 5a) and Binding Site II (Fig. 5b) with similar total binding energies. The binding of FapC on SPT in Binding Site I is mediated in two distinct binding regions, the first in the non-repeat helical segment, and the second in the three imperfect R3 repeat sequences of FapC stacked on top of each other, along with the additional β-strands linker region (only the β-sheets connecting the R3 repeat sequences) formed inside the amyloid core (Fig. 5a). The first binding region of FapC on site I is revealed to be in contact with the C–terminal region of SPT and especially with α–helix G (the only helix in the C–terminal region) and the loops connecting the three–stranded antiparallel β–sheets β20, β21, and β22. As for the second region, it is found in contact with part of the N–terminal proteolytic domain of SPT and especially with α–helix B and the coils linking helices A and B and helix B with β–sheet β1. All binding contacts between SPT and FapC are mediated by residues belonging to the R3 repeat sequence of FapC and the helical region. The binding of FapC on SPT in Binding Site II includes the catalytic active site cleft ligated by three histidine residues Hl76, Hl80, and Hl86, and one tyrosine, Y216, completing thus the catalytic tetrad (Supplementary Table S1 and Fig. 5b).

The binding interface region between FapD and SPT is in the active catalytic center of FapD, indicating a possible proteolytic interference in the active peptidase function of FapD, beyond its processing chaperone activity (Fig. 6). Binding of SPT to FapD affects the proteolytic modification of FapC exerted by FapD while residing in the periplasmic space between the inner and outer membrane, before its secretion from the cell.

## Discussion

The emergence of the so-called “superbugs” is potentially the “Phantom Menace” of future medicine. The extensive use of antibiotics and antibacterial agents in the past has raised a whole generation of multi-resistant bacterial strains, which will probably dominate the interest of many future generations of researchers and clinical practicians. These strains will allegedly cause a plethora of impossible-to-treat infection cases, especially inside the nosocomial environment, that will make the public expenses for healthcare and mortality rates explode to unprecedented heights (Dadgostar 2019). Consequently, it is of imperative need to implicate novel medications against microbial agents other than antibiotics, in order to minimize the employed doses, if not avoid their use at all (Bush et al. 2011).

One of the mechanisms for the multi-gaining of antibiotic resistance is tragically the most frequent mode of microbial growth: biofilms. Biofilm-growing bacteria cause chronic infections, characterized by persistent inflammation and tissue damage, while actively obstructing antimicrobial compounds and the host immune system. Notably, biofilm-growing bacteria may be up to 100- to 1000-fold more resistant to antibiotics, in comparison with planktonic bacteria (Høiby et al. 2010). The EPS of biofilms is actively contributing to resistance acquisition, by neutralizing or diluting antimicrobial substances. At the same time, biofilms can thrive through slow growth and the building of defences that create more and more resistant clones. Nutrient depletion and reduced oxygen levels may also contribute to the matter (Percival et al. 2015).

In the current study, *P. aeruginosa* biofilms have been studied. *P. aeruginosa* is one of the core species at HAI, characterized naturally by numerous resistances (ECDC 2019). Also, *P. aeruginosa* is the main pathogen for CF patients, who suffer from chronic infections with poor prognosis (Lyczak et al. 2002). *P. aeruginosa* in CF lungs forms biofilms with bacteria that are characterized by very slow growth (Yang et al. 2008). As antibiotics are usually active against doubling *P. aeruginosa* cells, their administration is generally failing to protect patients suffering from biofilm infections (Høiby et al. 2010). Thus, in the current study, the employment of an alternative anti-biofilm agent, namely SPT, has been attempted. SPT has been previously proven to be able to block or eradicate in vitro and/or in vivo biofilm formation from several bacteria, including *Listeria monocytogenes* (Longhi et al. 2008), *Staphylococcus aureus* (Artini et al. 2013; Papa et al. 2013; Selan et al. 2015, 2017; Devlin et al. 2021; Katsipis and Pantazaki 2023), and *Staphylococcus epidermidis* (Mecikoglu et al. 2006; Artini et al. 2013). The anti-biofilm effect of SPT on *P. aeruginosa* has not gained enough attention at present, with very few studies referencing its potential clinical employment (Selan et al. 1993; Passariello et al. 2012; Artini et al. 2022).

The anti-biofilm capacity of SPT against *P. aeruginosa* Boston 41501 (ATCC 27853) is demonstrated here—to the best of our knowledge—for the first time. Boston 41501 is a model strain isolated from a blood infection, presenting resistance against several antibiotic agents and disinfectants and employed commonly in studies of antibiotic resistance (Brown et al. 1995). In the current study, SPT was able to inhibit biofilm formation from the specific *P. aeruginosa* strain at both plastic and glass surfaces in a significant manner. Examination with confocal microscopy of the biofilms grown on glass has also revealed a much thicker and denser matrix, in comparison with biofilms grown in the presence of SPT.

In a recent study, Artini et al. have also confirmed the anti-biofilm efficacy of SPT against *P. aeruginosa* PA14 and several isolated clinical strains from CF lungs, impairing biofilm formation vaguely 40 to 70%. The authors proved that under the effect of SPT, *P. aeruginosa* presented impaired attachment to abiotic surfaces as well as reduced ability for adhesion/invasion of eukaryotic cells. Also, treatment with SPT decreased the virulence of the bacteria, as confirmed by altered pyocyanin and pyoverdine production, impaired swarming motility, and reduced staphylolytic protease production (Artini et al. 2022). However, the authors did not study the possible correlation of these parameters with biofilm formation and did not provide a possible mechanistic explanation for biofilm inhibition. Selan et al. worked on a combinational therapy with SPT and ofloxacin for several clinical strains of *P. aeruginosa* isolated from prosthetic device infections. The results proved that SPT could assist the microbiocidal effect of the employed antibiotic against both biofilm and planktonic bacteria (Selan et al. 1993). Several other studies also offered insights into the process, providing evidence that SPT can also enhance the activity of ofloxacin, azithromycin, ciprofloxacin, vancomycin, rifampicin and levofloxacin against *S. aureus, S. epidermidis,* and methicillin-resistant *S. aureus* (MRSA) (Selan et al. 1993; Thaller et al. 1997; Hogan et al. 2017; Gupta et al. 2017). Additionally, SPT was previously found to be able to be employed as a combined therapy of peri-implantitis, enhancing the success rates and providing better tissue repair around successfully treated tooth implants (Sannino et al. 2013).

Interestingly, our results have demonstrated that SPT can suppress biofilm build-up more strongly in glass slides (hydrophilic surface) than in plastic surfaces of TCP plates (hydrophobic surface), presenting an IC_50_ of 0.27 µg/mL and 11.26 µg/mL, respectively. Pereira et al. have verified that sessile growth of *P. aeruginosa* in plastic or glass surfaces induces differential biofilm phenotypes, with biofilms growing on plastic maturing at a faster pace than the ones in glass, with the latter presenting a more immature structure. The authors suggest that charge, hydrophobicity, and roughness of the surface would be important factors during biofilm development (Pereira et al. 2015). Additionally, in a multi-microbial biofilm study, *P. aeruginosa* showed comparatively higher hydrophobic surface properties than *E. coli* and *S. aureus*, which was regarded as significant for bacterial adhesion and directly proportional to biofilm formation (Mirani et al. 2018). Thus, it could be deduced that the highly hydrophobic area of TCP wells would strongly support steadier biofilm formations from *P. aeruginosa*, which could explain the need for significantly higher titers of SPT for obstructing its settling.

Low oxygen and nutritional requirements, slow growth, extended survival and antibiotic resistance gaining are recognized in *P. aeruginosa*. These conditions are mainly found in CF patient lungs, where bacteria thrive in a biofilm mode (Rossi et al. 2021). In the current study, the ability of *P. aeruginosa* to present increased levels of biofilm formation under nutritional deficiency has been verified. Previous reports demonstrate that glucose supports the growth of small, structured biofilms of *P. aeruginosa* than uniform biofilms, including less motile and swarming bacteria—a behavior attributed to quorum sensing control. In addition, *P. aeruginosa* grown with glucose express higher amounts of rhamnolipids—a surfactant that leads to biofilm dispersion and remodeling (Shrout et al. 2006), suggesting that in nutrient-rich environments, *P. aeruginosa* would preferably adopt a motile growth pattern.

To further study the effect of SPT, *P. aeruginosa* grown under the effect of SPT was examined for its metabolic viability. Results proved that SPT could ameliorate bacterial viability, in a way that coincides with biofilm inhibition, as also implied by correlation analysis. Recently, treatment of *S. aureus* biofilms with SPT has provided evidence for dysregulation of phosphate metabolism—a crucial factor for biofilm formation and bacterial viability (Danikowski and Cheng 2018; Katsipis et al. 2021; Katsipis and Pantazaki 2023). However, as far as we know, this is the first time that the effect of SPT on the viability of *P. aeruginosa* or any other Gram-negative bacteria is assessed with a standard method as the MTT test. Previously, Artini et al. have also proposed that impaired production of pyocyanin and pyoverdine due to SPT treatment is an effect correlating with reduced virulence—a factor the authors regard more important than viability inhibition (Artini et al. 2022). It should however be noted that the MTT assay used here is a rough measure of the bacterial metabolic activity, basically due to oxidoreductive enzymes (Grela et al. 2018). So, these results also provide evidence that SPT treatment may lead to impairments in bacterial homeostatic mechanisms that coordinate metabolism—an event forcing bacteria to abandon the biofilm environment. It is worth mentioning that SPT did not seem to impair the planktonic bacteria titers, as calculated by turbidity assays, and thus its possible toxic behavior seems not to be applied on motile than to sessile, biofilm-forming bacteria. However, it should be taken under notice that turbidity measurements cannot exclude dead bacteria. To that end, part or whole of the sum of the metabolically inactive bacteria may correspond to irreversibly non-viable cells. In a previous study of combinational therapy of several *P. aeruginosa* clinical strains with ofloxacin, SPT significantly reduced the minimal inhibitory concentration and minimal bactericidal concentration of the employed antibiotic, only regarding sessile bacteria, proving the thought that SPT specifically targets biofilm-forming bacteria (Selan et al. 1993). A following study assessing the ability of *P. aeruginosa* to grow new biofilms or colonies after SPT employment, would give more insight into the possible immediate and independent toxicity of this protease.

The functional amyloid titers, as estimated with CR retainment, seem to correlate with increased biofilm formation under low nutrition in the current study, as previously demonstrated for curli expression in several *Enterobacteriaceae* (Castelijn et al. 2012; Paytubi et al. 2017; Leech et al. 2020). Studies on the function of *P. aeruginosa* Fap and its expression are still in their first steps (Dueholm et al. 2010, 2013; Zeng et al. 2015). Fap expression is imperative for bacterial adaptation in CF lungs and biofilm formation and its overexpression is enough to cause a similar phenotype of lung infection by *P. aeruginosa* in mice non-carrying the CF-related allele (Beg et al. 2023). Also, Fap has been proven to increase the hydrophobicity and stiffness of *Pseudomonas* biofilms, providing better resistance against environmental stress, and providing better chances for successful colonization. Deletion of most of the Fap operon genes leads to the loss of these characteristics (Zeng et al. 2015). Also, *FapC* deletion – the main component for ALF buildup, impedes the virulence of *P. aeruginosa*, while deletion of other operon genes (except *FapA*), leads to lower aggregation and diminished biofilm formation (Dueholm et al. 2013).

Reduced amyloid titers in *P. aeruginosa* biofilms due to SPT treatment were verified in the current study, with both CR-retainment methodology and by observing and analyzing the ThT-binding of amyloids with fluorescence microscopy. As expected, attenuation of biofilm in the presence of SPT was firmly correlated with CR-staining, indicating that SPT could impair the early formation of ALF. The exact mechanism of this effect is yet to be discovered. It was previously demonstrated that SPT could impair the amyloid formation of insulin fibers—a model for anti-amyloid studies both in vitro and in vivo (Metkar et al. 2017, 2020). A proteolytic activity against polymerizing ALF could be expected, as SPT was previously reported to affect several extracellular peptides of *S. aureus*, in accordance with biofilm inhibition (Artini et al. 2011). However, a later report proved that the SPT effect on *S. aureus* biofilm could not be attributed to a proteolytic mechanism, as mutations affecting the active site of the enzyme did not impair its anti-biofilm efficacy (Selan et al. 2015).

In silico molecular docking studies also provided mechanistic insight into the understanding of the interaction between SPT and FapC or FapD, and demonstrated a possible binding/proteolytic activity of SPT on both peptides. During the fibril build-up, each FapC monomer is stabilized not only by interactions with other monomers in the fibril, but also by contacts between the individual imperfect repeats, which most likely constitute β–hairpin helices (Rouse et al. 2017). R3 repeats are indicated to play an important role in stabilizing the fibrils (Christensen et al. 2020). Interestingly, there is evidence that repeat sequences are the main driving forces of the amyloidogenicity of FapC (Rasmussen et al. 2019). Remarkably, a mutant FapC lacking all three amyloidogenic conserved imperfect repeats R1–R3 (and thus only consisting of N– and C–terminal tails, as well as the two linker regions), is still able to fibrillate but at very slow and stochastic rates and with a greatly increased tendency to fragment, showing a secondary role for the linker regions in the aggregation process. Actually, in our study, several residues residing in the linker regions are revealed, as Q227, N225, A223, E186, A187, and S189. This implies a possible role of the linker binding contacts of FapC with SPT in the impairment of the fibrilization process. These results suggest that the formation of the FapC fibril component/SPT protein complex in the extracellular space may possibly affect the FapC fibril biogenesis. As regards to the role of FapD is still a little unclear, though it has essential proteolytic activity necessary for FapC secretion (Rouse et al. 2017).

To summarize, the results of this study verify the anti-biofilm effect of the protease SPT—a natural-fermenting product with no adverse effects, against *P. aeruginosa* ATCC 27853, regarding both plastic and glass surfaces. The anti-biofilm effect of SPT was verified with the TCP method and by employing both optical and confocal microscopy. Additionally, this effect is interrelated with decreased titers of functional amyloids, as estimated by the employment of amyloid-specific dyes CR and ThT, and of lowered viability/metabolic activity of bacteria. In silico studies provided mechanistic details on the possible binding of FapC and FapD peptides by SPT and suggested a probable proteolytic mechanism for their clearance. Future studies should now be aiming for analyzing the effect of SPT on *Pseudomonas* amyloids and verify its possible proteolytic role and the exact binding mechanism on Fap peptides. Furthermore, a molecular explanation for viability inhibition based on studying various physiological pathways should be pursued. A well-organized in vivo study on lab animals with CF should also be attempted, in order to further elucidate the employment of SPT in clinical practice. Whatsoever, the present results will hopefully resurface the possible employment of SPT in medicine as a counter against bacterial biofilms and in combination with antibiotic therapy, to minimize the dose of the latter and thus stop the evolution of more multi-resistant strains – especially in healthcare facilities- and for the manifestation of chronic infections, like the ones in CF patients.

### Supplementary Information

Below is the link to the electronic supplementary material.

Supplementary file1 (PDF 522 KB)

## Data Availability

The datasets generated during and/or analyzed during the current study are available from the corresponding author upon reasonable request.
