# Supplementary material for: In vitro and in silico evaluation of the serrapeptase effect on biofilm and amyloids of Pseudomonas aeruginosa
Source: Appl Microbiol Biotechnol. 2023 Sep 23;107(23):7269–85. doi: 10.1007/s00253-023-12772-1 (PMC10638192; doi:10.1007/s00253-023-12772-1)
Supplement: Supplementary file 1 — Supplementary file1 (PDF 522 KB) [file 253_2023_12772_MOESM1_ESM.pdf]

Supplementary Information

In vitro and *in silico* evaluation of the Serrapeptase effect on biofilm and amyloids of *Pseudomonas aeruginosa*

Applied Microbiology and Biotechnology

Georgios Katsipis[1,2], Dimitrios I. Avgoulas[2,3], George D. Geromichalos[4], Maria Petala[5], Anastasia A. Pantazaki[1,2*]

[1] Laboratory of Biochemistry, Department of Chemistry, Aristotle University of Thessaloniki, 54124 Thessaloniki, Greece

[2] Center for Interdisciplinary Research and Innovation, Laboratory of Neurodegenerative Diseases (LND), 57001, Thermi, Thessaloniki, Greece

[3] Laboratory of Chemical and Environmental Technology, Dept. of Chemistry, Aristotle University of Thessaloniki, 54 124, Thessaloniki 54124, Greece

[4] Department of General and Inorganic Chemistry, Faculty of Chemistry, Aristotle University of Thessaloniki, GR–54124 Thessaloniki, Greece

[5] Laboratory of Environmental Engineering & Planning, Department of Civil Engineering, Aristotle University of Thessaloniki, Thessaloniki 54 124, Greece

***Corresponding author:** Professor A.A. Pantazaki, Lab. of Biochemistry, Dept. of Chemistry, Aristotle University of Thessaloniki, 54124 Thessaloniki, Greece, Tel: +30-2310-997838 & Fax: +30-2310-997689

e-mail: natasa@chem.auth.gr,

Web: https://www.chem.auth.gr/staff/pantazaki/

Index

**Content**	**Page**
**S1. Pilot tests for the composition of the employed medium for biofilm formation**	3
**Fig. S1** Biofilm formation, planktonic bacteria growth, and functional amyloid levels of *Pseudomonas aeruginosa* ATCC 27853 with several TSB dilutions as growth medium.	3
**S2. Confocal microscopy of *Pseudomonas aeruginosa* biofilms on glass slides**	4
**Fig. S2** Effect of SPT dose on arithmetical mean height and developed area of biofilm covered surface.	4
**Fig. S3** Effect of SPT dose on the maximum height of peaks obtained on the biofilm covered surface.	5
**S3. *In silico* computational methods (molecular docking calculations)**	6
**Fig. S3** Proposed structural prediction models of two Fap fibril biogenesis, the major Fap amyloid component FapC amyloid–like fimbriae protein	6
**Table S1.** Binding interactions of the interface contact residues between SPT and the BLAST sequence similarity search by UniProt model of FapC (AF-C4IN70-F1-model_v4.pdb) at Binding Sites I and II.	7
**Table S2.** Binding interactions of the interface contact residues between SPT and FapD (modeled after the homologous C39 peptidase domain of ABC transporter PCAT1, PDB ID: 4RY2).	8
**Fig. S5** Predicted aligned error (PAE) plot for FapC amyloid-like fimbriae protein.	9

Section S1. Pilot tests for the composition of the employed medium for biofilm formation

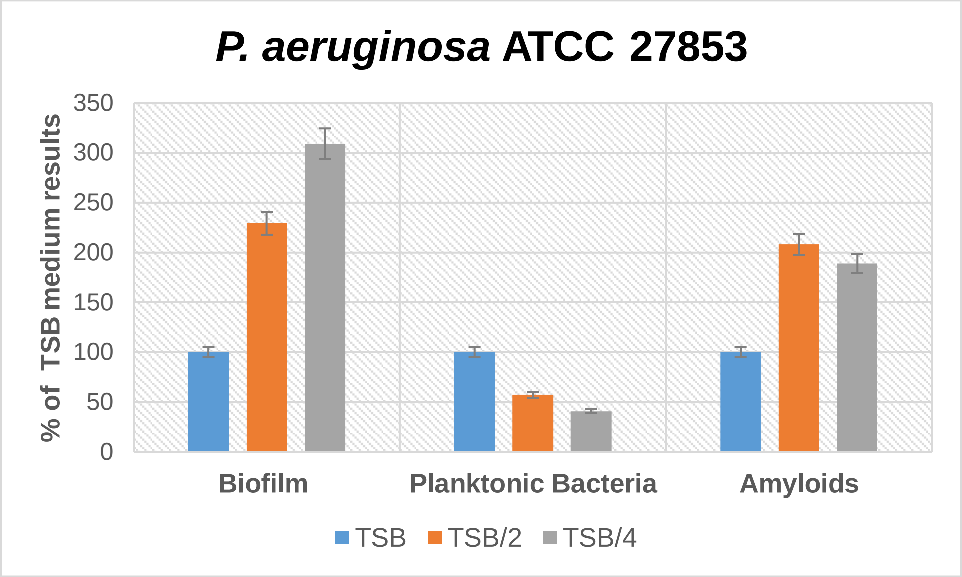

Supplementary Fig. S1 Biofilm formation, planktonic bacteria growth, and functional amyloid levels of *Pseudomonas aeruginosa* ATCC 27853 with several TSB dilutions as growth medium. Bacteria were grown in several dilutions of tryptic-soy broth (TSB) medium, under stative conditions in 96-well tissue culture plates, for 24h and 37°C. After incubation, planktonic bacteria were determined with their turbidity at 630 nm and biofilm formation was semi-quantified with crystal violet. Functional amyloid levels were determined inversely with Congo red. Bars represent % of the values received from standard TSB medium cultures. Bars represent mean values ± standard error of means from at least three independent experiments.

Section S2. Study of *P. aeruginosa* biofilms with confocal microscopy

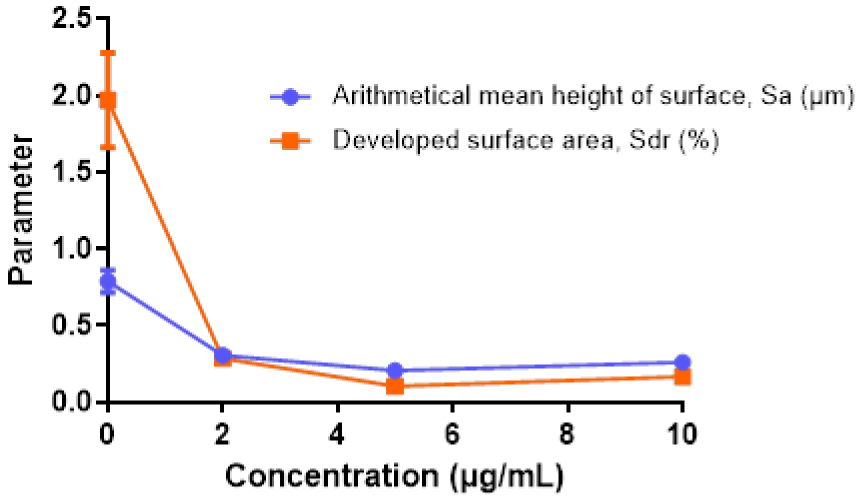

Supplementary Fig. S2 Effect of SPT dose on arithmetical mean height and developed area of biofilm covered surface.

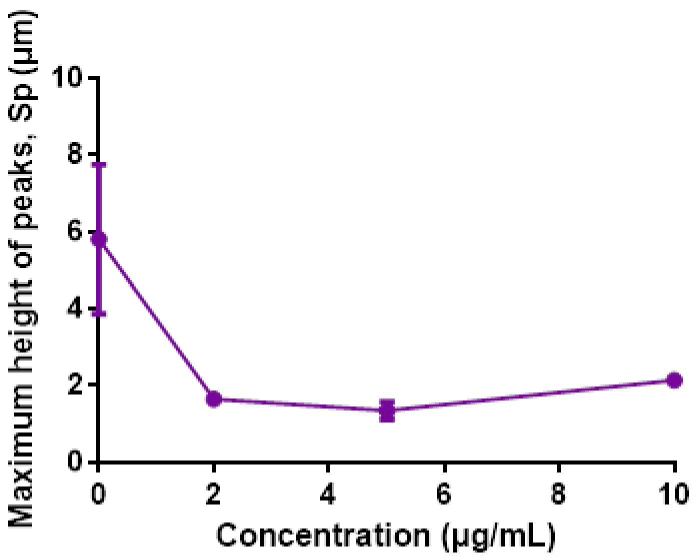

Supplementary Fig. S3 Effect of SPT dose on the maximum height of peaks obtained on the biofilm covered surface.

Section S3. *In silico* computational methods (molecular docking calculations)

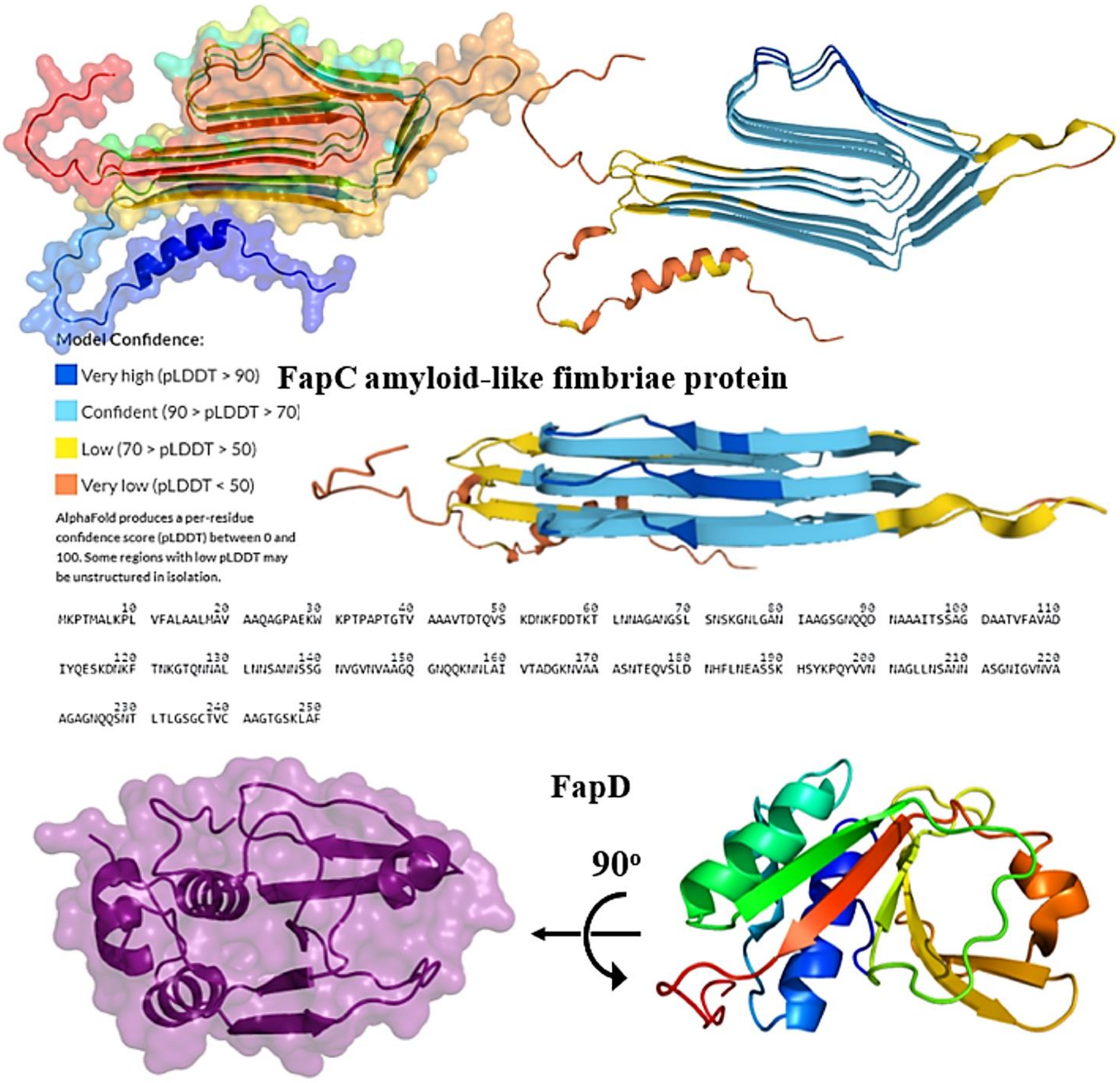

Fig. S4 Proposed structural prediction models of two Fap fibril biogenesis, the major Fap amyloid component FapC amyloid–like fimbriae protein produced by the BLAST sequence similarity search by UniProt model of FapC (AlphaFold Protein Structure Database, AF-C4IN70-F1-model_v4.pdb) illustrating also its protein sequence (the repeat sequences R1, R2, and R3 shown in the upper left panel in yellow, green, and red, respectively, are stacked on top of each other, while the linker regions seem to form additional β–strands inside the amyloid core), and the FapD modeled after the homologous C39 peptidase domain of ABC transporter PCAT1 (PDB ID: 4RY2), since there is no putative structural data in the literature (the Fap operon is not well characterized). Both proteins are illustrated either as cartoon alone colored by spectrum and secondary structure or with additional depiction of semi–transparent surface.

Table S1. Binding interactions of the interface contact residues between SPT and the BLAST sequence similarity search by UniProt model of FapC (AF-C4IN70-F1-model_v4.pdb) at Binding Sites I and II. Atom numbering and bond lengths are derived from PyMol software (type of interaction: H-b: hydrogen bond, π-π: pi-pi displaced and T-shaped, π-alkyl: pi-alkyl hydrophobic, π-anion: pi-negative charged electrostatic interactions, π-polar: pi-polar, H-ph: hydrophobic, Polar).

**SPT / FapC protein-protein complex**							
**Biding Site I**				**Biding Site II**			
**SPT**	**FapC**	**Bond length (Å)**	**Type**	**SPT**	**FapC**	**Bond length (Å)**	**Type**
T166/OH	L97/O	2.9	H-b	H176/Nε2	P26/Cβ	2.9	Polar
T166/Cγ2	F100	2.5	π-alkyl	H180/Nε2	P26/Cγ	3.2	Polar
T166/Oγ1	F100	3.6	π-polar	H186/Nε2	P26/Cβ	2.7	Polar
T166/O	A98/NH	3.4	H-b	Y216/OH	A27/N	2.5	H-b
D168/NH	Q51/O	3.2	H-b	Y216	A27/Cβ	3.7	π-alkyl
D168/Oδ1H	Q51/O	2.8	H-b	Y216	A27/O	3.1	π-polar
D168/Oδ1H	Q51/N	3.4	H-b	Y169/OH	E28/Oε1	2.5	H-b
D168/Oδ1H	T50/O	3.2	H-b	Q133/Oε1	E28/Oε2	3.2	H-b
H299	L18/O	3.7	π-polar	Q133/Cβ	E28/Cβ	2.9	H-b
H299	L18/Cβ	2.8	π-alkyl	T132/Cα	E28/O	3.2	Polar
H299	D17/Oδ1	2.9	π-anion	A134/O	P26/O	2.8	Polar
K278/O	R96/NH2	2.2	H-b	E177/Oε2	P26/Cγ	2.3	Polar
K278/O	R96/Nε	2.4	H-b	F137	A22/O	3.8	π-polar
S276/O	L18/Cδ2	2.2	Polar	F137	Q23/Nε2	2.8	π-polar
K278/Cβ	L18/Cδ1	3.2	H-ph	T141/Cγ2	A22/Cβ	3.6	H-ph
V279/Cγ1	R96/NH2	3.9	Polar	I142/O	A19/Cβ	2.1	Polar
S271/O	N95/Nδ2H	3.5	H-b	W143	V20/Cγ2	3.1	π-alkyl
Q277/Oε1	N95/Cβ	2.1	Polar	W143	A16/O	3.9	π-polar
Q277/Oε1	D94/O	3.2	Polar	W143	Q23/Oε1	3.8	π-polar
D268/O	N95/O	2.6	Polar	W143	N167/Oδ1	3.9	π-polar
R171/Cβ	L97/Cδ1	3.2	H-ph	G195/O	W30/Nε1H	2.0	H-b
Q88/Oε1	L97/Cδ2	3.4	Polar	E194/Oε2	W30	3.1	π-anion
S276/OγH	Q15/O	2.2	H-b	G131/Cε	K31/Cε	3.9	H-ph
Q277/Cγ	R96/Cγ	2.8	H-ph	G131/O	K29/Cγ	3.2	Polar
T273/Cγ2	N95/Nδ2	3.6	Polar				
Y230	T50/Oγ1	2.1	π-polar				
G227/O	D17/Oδ2H	3.0	H-b				
H229	T19/Cγ2	3.7	π-alkyl				
G228/O	T50/OH	2.1	Polar				
Y169	Q51/Nε2	3.9	π-polar				
Q88/Nε2	I92/Cγ2	3.0	Polar				
K278/Cγ	D17/Oδ1	3.2	Polar				
G228/O	T19Cγ2	3.5	Polar				

Table S2. Binding interactions of the interface contact residues between SPT and FapD (modeled after the homologous C39 peptidase domain of ABC transporter PCAT1, PDB ID: 4RY2). Atom numbering and bond lengths are derived from PyMol software (type of interaction: H-b: hydrogen bond, π-π: pi-pi displaced and T-shaped, π-alkyl: pi-alkyl hydrophobic, π-anion: pi-negative charged electrostatic interactions, π-polar: pi-polar, H-ph: hydrophobic, Polar).

SPT / FapD C39 protein-protein complex			
**SPT**	**FapD C39**	**Bond length (Å)**	**Type**
T166/OH	L97/O	2.9	H-b
T166/Cγ2	F100	2.5	π-alkyl
T166/Oγ1	F100	3.6	π-polar
T166/O	A98/NH	3.4	H-b
D168/NH	Q51/O	3.2	H-b
D168/Oδ1H	Q51/O	2.8	H-b
D168/Oδ1H	Q51/N	3.4	H-b
D168/Oδ1H	T50/O	3.2	H-b
H299	L18/O	3.7	π-polar
H299	L18/Cβ	2.8	π-alkyl
H299	D17/Oδ1	2.9	π-anion
K278/O	R96/NH2	2.2	H-b
K278/O	R96/Nε	2.4	H-b
S276/O	L18/Cδ2	2.2	Polar
K278/Cβ	L18/Cδ1	3.2	H-ph
V279/Cγ1	R96/NH2	3.9	Polar
S271/O	N95/Nδ2H	3.5	H-b
Q277/Oε1	N95/Cβ	2.1	Polar
Q277/Oε1	D94/O	3.2	Polar
D268/O	N95/O	2.6	Polar
R171/Cβ	L97/Cδ1	3.2	H-ph
Q88/Oε1	L97/Cδ2	3.4	Polar
S276/OγH	Q15/O	2.2	H-b
Q277/Cγ	R96/Cγ	2.8	H-ph
T273/Cγ2	N95/Nδ2	3.6	Polar
Y230	T50/Oγ1	2.1	π-polar
G227/O	D17/Oδ2H	3.0	H-b
H229	T19/Cγ2	3.7	π-alkyl
G228/O	T50/OH	2.1	Polar
Y169	Q51/Nε2	3.9	π-polar
Q88/Nε2	I92/Cγ2	3.0	Polar
K278/Cγ	D17/Oδ1	3.2	Polar
G228/O	T19Cγ2	3.5	Polar

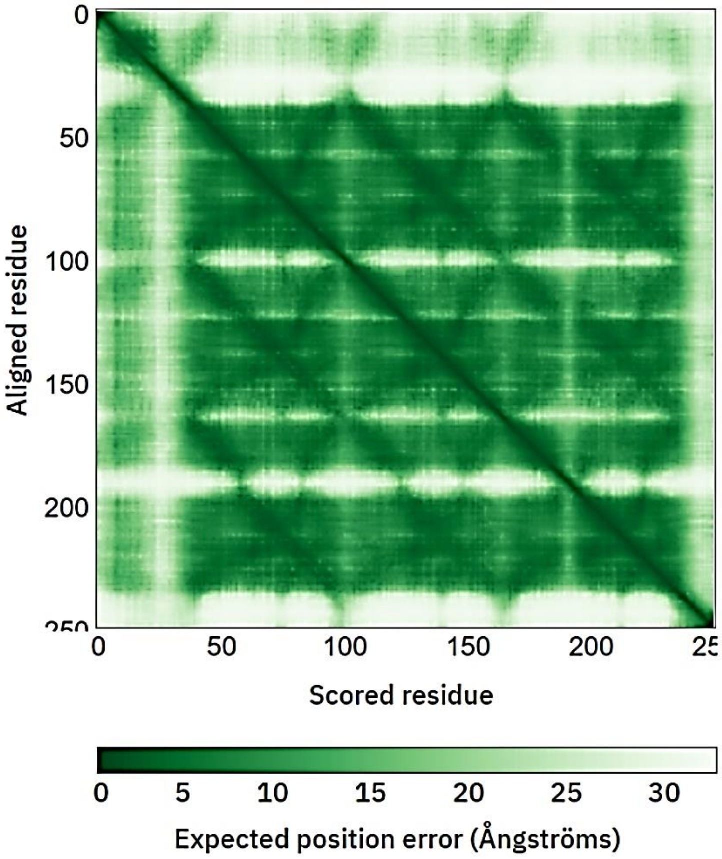

Fig. S5 Predicted aligned error (PAE) plot for FapC amyloid-like fimbriae protein. The color at position (x, y) indicates AlphaFold’s expected distance position error at residue x, when the predicted and true structures are aligned on residue y. Note that the PAE plot is not an inter-residue distance map or a contact map. Instead, the shade of green indicates expected distance error in Ångströms. Dark green is good (low error), light green is bad (high error) (derived from https://alphafold.ebi.ac.uk/entry/C4IN70).
